# Supplementary material for: Naked-Eye Chromogenic Test Strip for Cyanide Sensing Based on Novel Phenothiazine Push–Pull Derivatives
Source: Biosensors (Basel). 2022 Jun 13;12(6):407. doi: 10.3390/bios12060407 (PMC9220876; doi:10.3390/bios12060407)
Supplement: Supplementary file 1 [file biosensors-12-00407-s001.zip › biosensors-1738715-supplementary.pdf]

Supporting Information

Naked-eye chromogenic test strip for cyanide sensing based on novel phenothiazine push-pull derivatives

Pedro E. Martín Várguez,† Jean-Manuel Raimundo*

Aix Marseille Univ, CNRS, CINAM, Marseille, France.

Table of Contents

^{1}H NMR, ^{13}C NMR compounds **1**, **6**, **7** and **8** 2

Determination of theoretical spectrum (Figure 12) for **8**.CN^-, **8**.NO_2^- and **8**.$CH_2CO_2^-$ ……...6

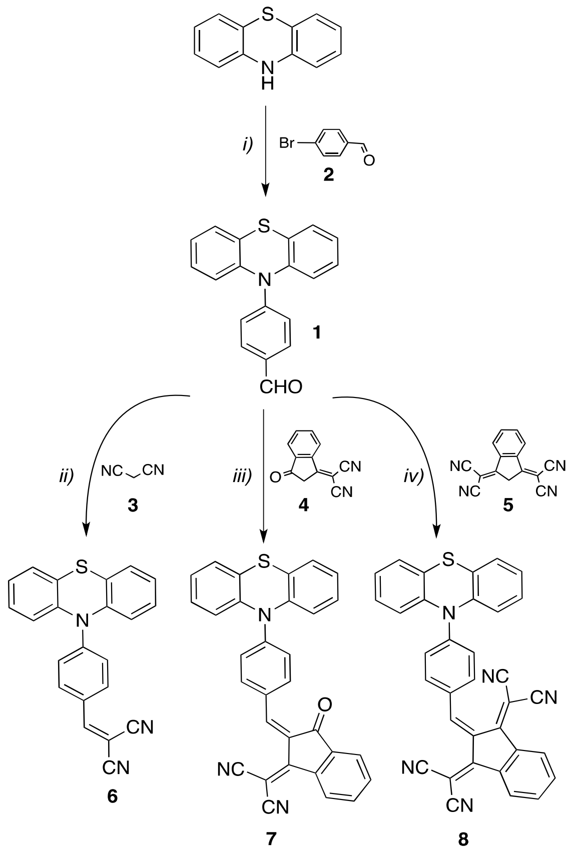

^{1}H and ^{13}C NMR – compound **1**

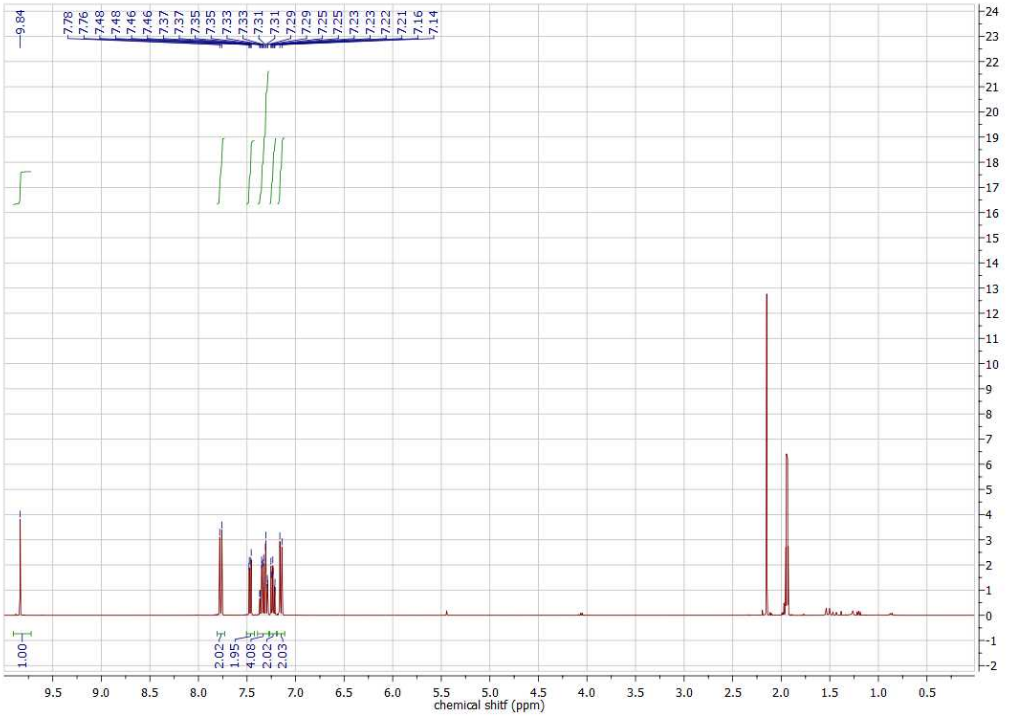

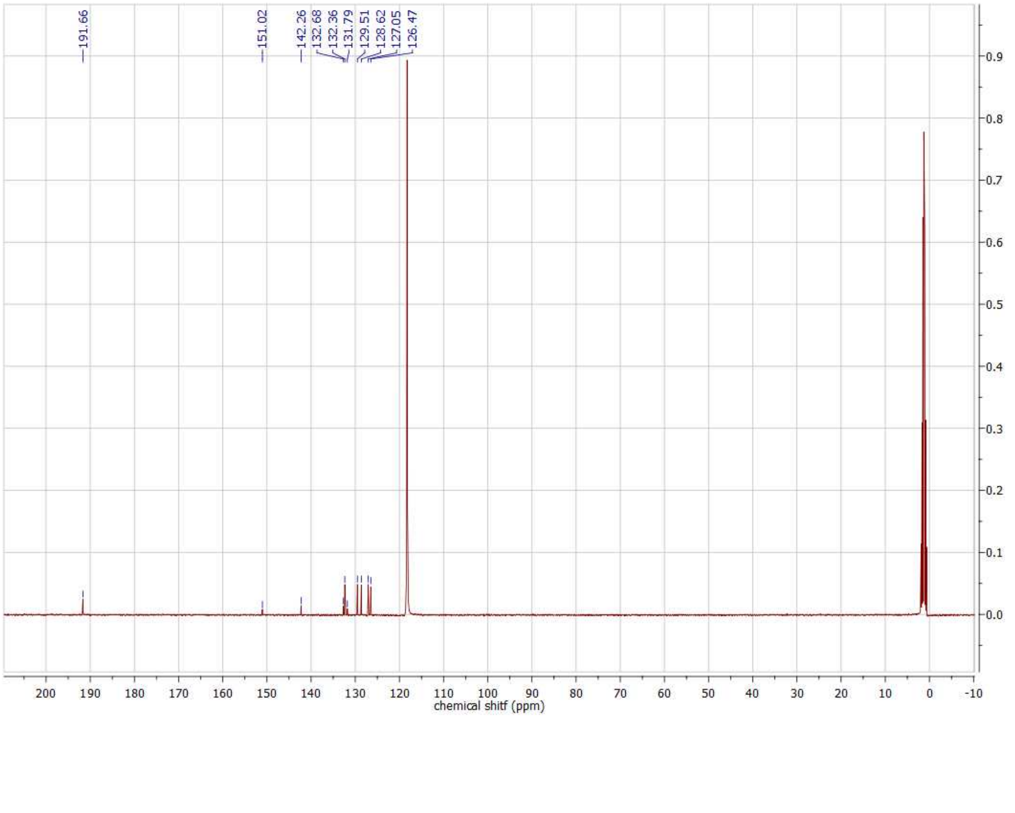

^{1}H and ^{13}C NMR – compound **6**

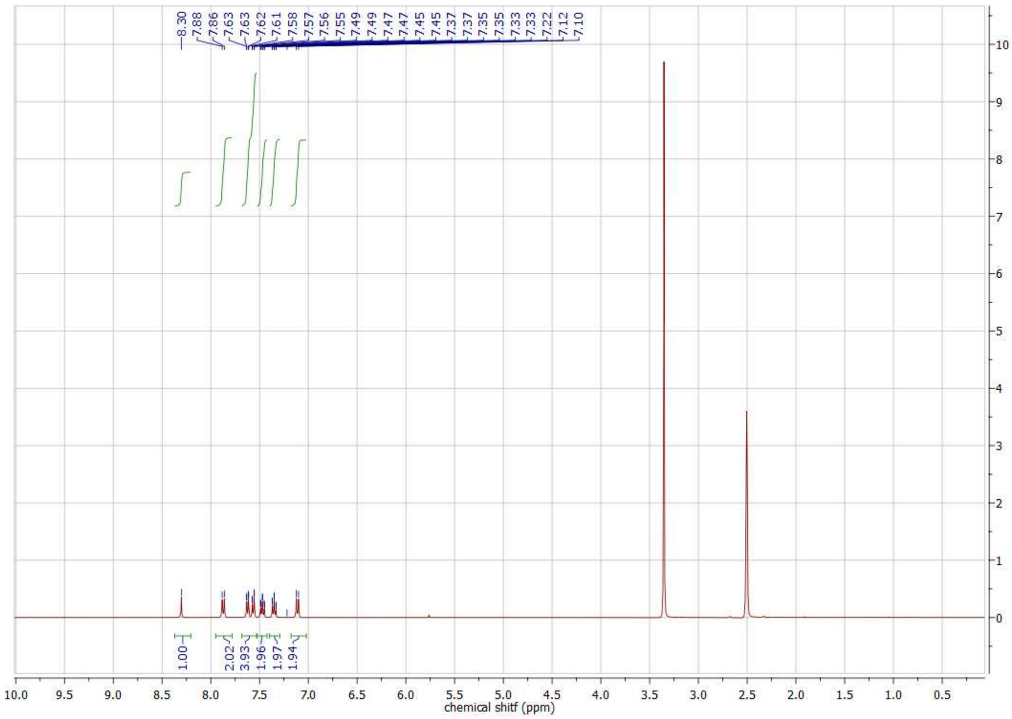

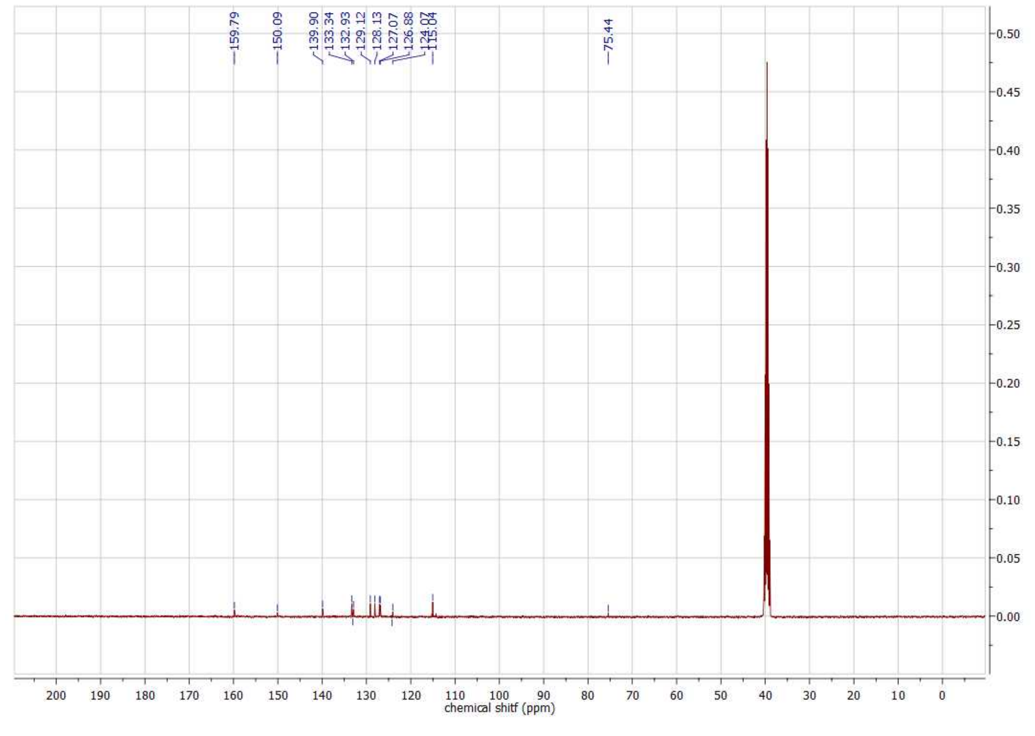

^{1}H and ^{13}C NMR – compound **7**

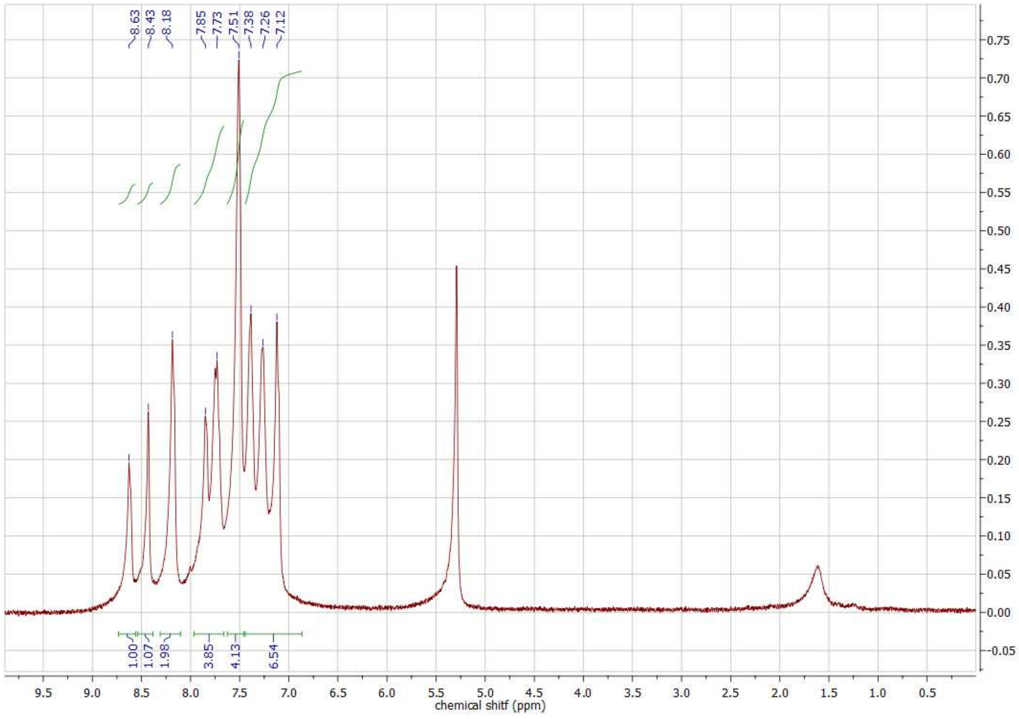

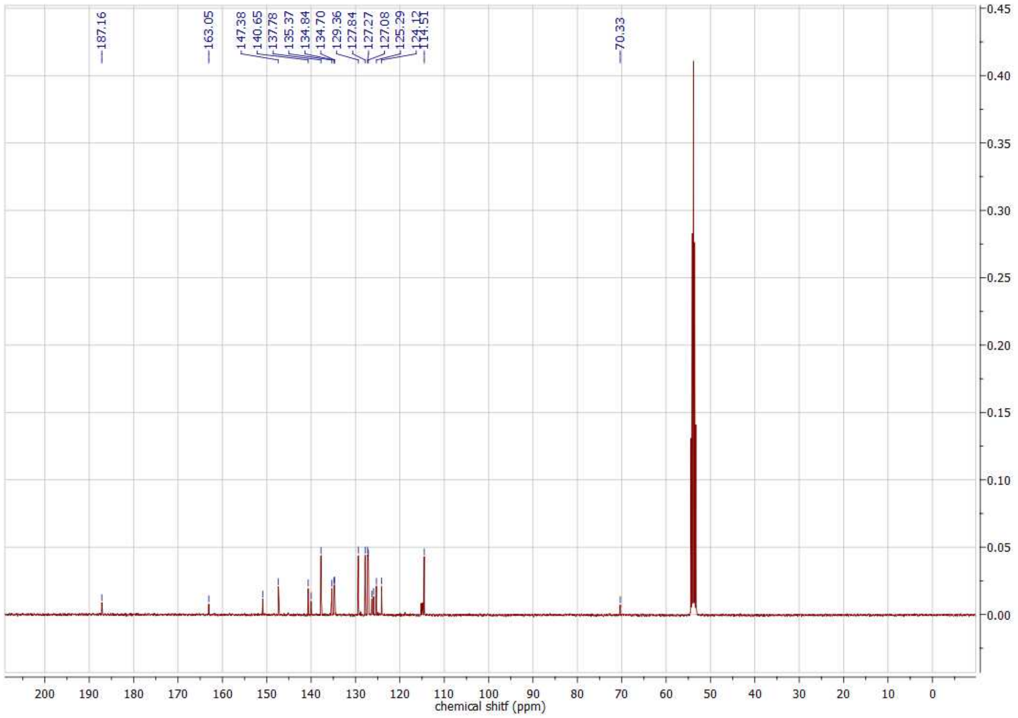

^{1}H and ^{13}C NMR – compound **8**

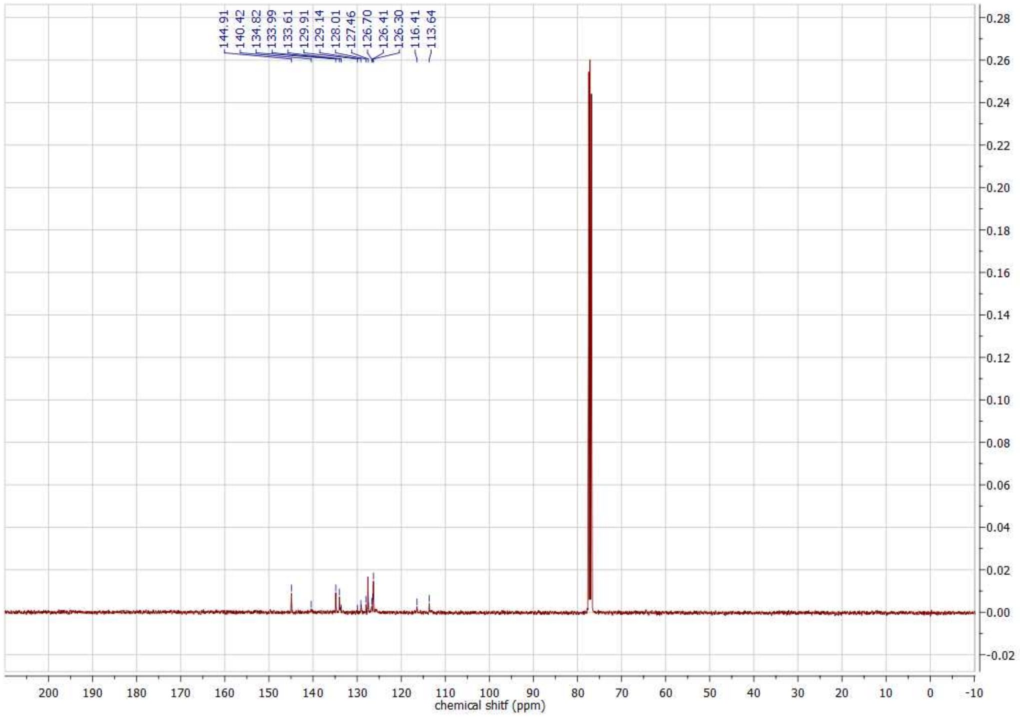

Determination of theoretical spectrum (Figure 12) for **8**.CN^-, **8**.NO_2^- and **8**.$CH_2CO_2^-$

Once the stoichiometries are established for **8**.CN^-, **8**.NO_2^- and **8**.$CH_2CO_2^-$, their theoretical spectra can be calculated from their experimental data. Interactions between molecules are neglected and the total absorbance corresponds to a linear combination of each chromophore present in solution, as given in **Eq.1**:

$$A^{exp} = \varepsilon_{8}[\mathbf{8}] + \varepsilon_{8.CN^-}[\mathbf{8}.CN^-] + \varepsilon_{8.NO_2^-}[\mathbf{8}.NO_2^-] + \varepsilon_{8.CH_2CO_2^-}[\mathbf{8}.CH_2CO_2^-] + \varepsilon_{8.(CH_2CO_2^-)_2}[\mathbf{8}.(CH_2CO_2^-)_2] \qquad \text{Eq. 1}$$

where ε_x represents the molar extinction coefficient and [x] the concentration of each specie in solution. [x] can be deduced from their molar concentration and their binding constant *K*. In **Eq. 1** only ε_8 is known while the others as well as their respective association constants remain unknown. In addition, we must assume that ε_x is only constant at a fixed wavelength. In order to conduct the calculation over *n* wavelengths it is necessary to fix 4*n* + 4 constants hence 4*n* + 4 equations. Experimentally, *3n* experiments were conducted in order to determine the interactions of compound **8** with each anion (CN^-, NO_2^- and $CH_2CO_2^-$), *3n* experiments when a complex **8**.X is formed with a specific anion and varying the others and *1n* experiments of **8** in presence of the 3 anions corresponding to a total of *7n* different equations. With a *n* = 227 from 425 to 700 nm , *7n* > *4n* + *n* and it is possible to determine the *4n* + *n* constants by the comparison of the experimental spectrum with the theoretical ones obtained by regression. As first approximation the spectrum (*4n*) and the binding constants can be set as equal to that of **8** and to 1, respectively. At the equilibrium, **Eq. 1** can be rewritten as follow :

$$A^{exp} = \varepsilon_{8}[\mathbf{8}]_{eq} + \varepsilon_{8.CN^-}[\mathbf{8}.CN^-]_{eq} + \varepsilon_{8.NO_2^-}[\mathbf{8}.NO_2^-]_{eq} + \varepsilon_{8.CH_2CO_2^-}[\mathbf{8}.CH_2CO_2^-]_{eq} + \varepsilon_{8.(CH_2CO_2^-)_2}[\mathbf{8}.(CH_2CO_2^-)_2]_{eq} \qquad \text{Eq. 2}$$

With,

$$[8]_0 = [8]_{eq} + [8.CN^-]_{eq} + [8.NO_2^-]_{eq} + [8.CH_2CO_2^-]_{eq} + [8.(CH_2CO_2^-)_2]_{eq} \qquad \text{Eq. 3}$$

$$[CN^-]_0 = [CN^-]_{eq} + [8.CN^-]_{eq} \qquad \text{Eq. 4}$$

$$[NO_2^-]_0 = [NO_2^-]_{eq} + [8.NO_2^-]_{eq} \qquad \text{Eq. 5}$$

$$[CH_2CO_2^-]_0 = [CH_2CO_2^-]_{eq} + [8.CH_2CO_2^-]_{eq} + 2[8.(CH_2CO_2^-)_2]_{eq} \qquad \text{Eq. 6}$$

$$K_{8.CN^-} = [8.CN^-]_{eq}/([8]_{eq}[CN^-]_{eq}) \qquad \text{Eq. 7}$$

$$K_{8.NO_2^-} = [8.NO_2^-]_{eq}/([8]_{eq}[NO_2^-]_{eq}) \qquad \text{Eq. 8}$$

$$K_{8.CH_2CO_2^-} = [8.CH_2CO_2^-]_{eq}/([8]_{eq}[CH_2CO_2^-]_{eq}) \qquad \text{Eq. 9}$$

$$K_{8.(CH_2CO_2^-)_2} = [8.(CH_2CO_2^-)_2]_{eq}/([8]_{eq}[8.CH_2CO_2^-]_{eq}) \qquad \text{Eq. 10}$$

Equilibrium concentration of $[\mathbf{8}]_{eq}$, $[CN^-]_{eq}$, $[NO_2^-]_{eq}$, $[\mathbf{8}.CN^-]_{eq}$, $[\mathbf{8}.CH_2CO_2^-]_{eq}$, $[\mathbf{8}.NO_2^-]_{eq}$, $[\mathbf{8}.CH_2CO_2^-]_{eq}$ and $[\mathbf{8}.(CH_2CO_2^-)_2]_{eq}$ are computed by solving the nonlinear system imposed by **Eqs. 3-10**, and within **Eq. 3** is possible to find a theoretical spectrum. Thus, the repression problem can be solved by minimizing the sum of squares of the residuals of the experimental set data. The calculations were performed with a Matlab script using the *lsqnonlin* function. The mathematical equations to find the free analytical concentration of each compound can be found elsewhere [45].

45. Thordason, P. Determining association constants from titration experiments in supramolecular chemistry. *Chem. Soc. Rev.* **2011**, *40*, 1305-1323.
